# Iatrogenic newborn weight loss: knowledge translation using a study protocol for your maternity setting

**DOI:** 10.1186/1746-4358-6-10

**Published:** 2011-08-15

**Authors:** Joy Noel-Weiss, A Kirsten Woodend, Dianne L Groll

**Affiliations:** 1 School of Nursing, University of Ottawa, 451 Smyth Road, Ottawa, ON, K1H 8M5, Canada; 2 Trent-Fleming School of Nursing, 1600 West Bank Drive, Peterborough, ON, K9J 7B8, Canada; 3 Department of Psychiatry, Queen's University, 752 King Street West, Kingston, ON, K7L 4X3, Canada

## Abstract

**Background:**

In our original study of newborn weight loss, we determined there were positive correlations among newborn weight loss, neonatal output, and the IV fluids mothers received before their babies' birth. Basically, an increase in maternal IV fluids is correlated to an increase in neonatal output and newborn weight loss. When assessing newborn weight change, our recommendation is to change baseline from birth weight to a weight measured at 24 hours. The purpose of this paper is to provide a protocol for clinicians to collect and analyze data from their own maternity site to determine if the newborns experience such an iatrogenic weight loss and to make decisions about how to assess newborn weight changes.

**Methods:**

We recommend a prospective observational study with data collected about maternal fluids, neonatal output, and newborn weight measurements. The methods we suggest include specifics about recruitment, data collection, and data analysis.

**Discussion:**

Quality assurance and research ethics considerations are described. We also share practical information that we learned from our original study. Ultimately, to encourage knowledge translation and research uptake, we provide a protocol and sound advice to do a research study in your maternity setting.

## Background

It is expected that breastfed newborns will lose weight following birth [1-4]. Clinicians (e.g., dieticians, lactation consultants, nurses, and physicians) who work with breastfeeding women hold a number of assumptions about newborn weight change including: (a) weight measured within minutes of birth is an accurate baseline for calculating weight loss; (b) weight loss from baseline is due to insufficient intake (a lack of milk supply or transfer); and (c) weight loss past a certain threshold requires intervention which is often formula supplements [1-5]. Contrary to these assumptions, the newborn's weight loss may not be due to a lack of intake, but may be the result of increased neonatal output [6-9].

There is research literature to support the proposition that some neonates are born with a fluid overload and that the resulting diuresis (i.e., correction of fluids) contributes to their weight loss [6-9]. The purpose of this paper is to provide a protocol for clinicians to collect and analyze data from their own maternity sites to determine whether newborns experience iatrogenic weight loss. This protocol is based on a research study that concluded that birth weight should not be baseline; rather a weight measured at 24 hours post birth should be baseline for assessing newborn weight change [9].

**Figure 1:**
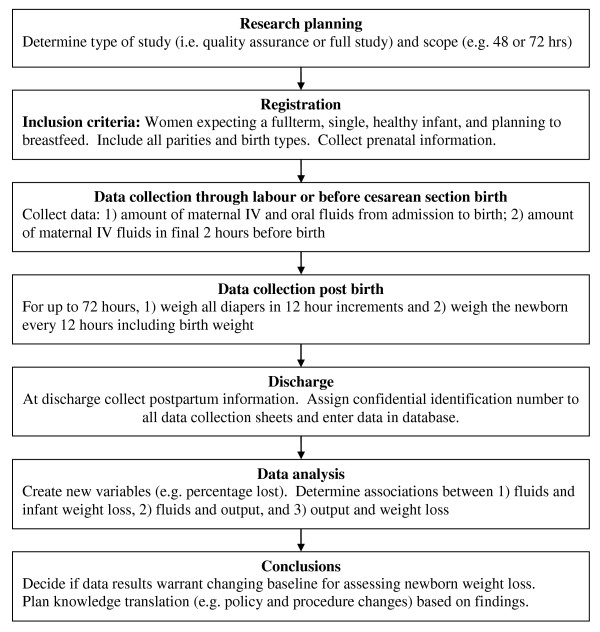
**Schematic of research design protocol**.

This protocol can be used to test three hypotheses: during the first 24-72 hours postpartum there are positive associations among (a) maternal fluids given during labour or prior to a caesarean section (intrapartum) and newborn weight loss; (b) intrapartum maternal fluids and neonatal output; and (c) neonatal output and newborn weight loss [9]. The protocol (see Figure 1) has been adapted from a study conducted by the authors [9].

### The original study

We conducted the prospective observational cohort study (N = 109) to explore associations between maternal fluids given during parturition, neonatal output, and newborn weight loss. Maternal IV and oral fluids were recorded during labour or before a caesarean section from admission to birth. Participants weighed their newborns every 12 hours for 72 hours, then newborn weight was measured daily from Day 4 to Day 14. Parents used the same scale from the postpartum unit to home, and babies were weighed unclothed. Parents weighed all output (i.e., diapers) in 24 hour increments for the first 72 hours (note: 12 hour increments would be better).

Average newborn weight loss, in our study, was similar to reports in the literature [10-12]. At 60 hours postpartum (point of maximum weight loss), mean loss was 237.2 grams (SD 98; n = 96, range 70-467 grams) and the mean percentage lost from birth weight was 6.57 (SD 2.51; n = 96, range 1.83-13.06%) [9]. When groups, based on maternal fluids, were compared (≤1200 mls [n = 21] versus > 1200 mls [n = 53]), newborns lost 5.51% versus 6.93% (p = 0.03), respectively [9].

We found a positive relationship between maternal IV fluids from admission to birth and newborn weight loss in grams at 60 hours (r(83) = 0.216, p = 0.050) and a positive relationship between maternal IV fluids received in the final 2 hours before birth and weight loss in grams at 60 hours (r(38) = 0.406, p = 0.011). Neonatal output at 24 hours was also correlated to the IV fluids given 2 hours before birth (r(42) = 0.383, p = 0.012). On Day 1, there was a positive relationship between output and grams of weight loss (r(96) = 0.493, p < 0.001) [9]. It appears neonates experience diuresis in the first 24 hours with a related weight loss.

Overall, the results indicate that maternal fluids before birth are related to neonatal output and newborn weight loss in the early postpartum period. It appears neonates experience varying degrees of diuresis in the first 24 hours, and the consequent weight loss is a correction. It also appears average weight loss at the point of maximum weight loss is related to maternal fluids given before birth. From the original study, we conclude that clinicians should reconsider using birth weight as baseline when assessing newborn weight loss [9]. We recommend using a weight measurement at 24 hours postpartum as the baseline for assessing newborn weight loss [9]. Due to variations in birth practices (e.g., the use of IV fluids) among different maternity settings, we developed this protocol for other clinicians to determine if our recommendation is warranted in their site and, thus, to increase knowledge translation.

### Knowledge translation

Knowledge translation is known by several terms including knowledge transfer, dissemination, or research utilization [13]. The Canadian Institutes of Health Research defines knowledge translation as a "dynamic and iterative process that includes synthesis, dissemination, exchange and ethically sound application of knowledge to improve the health of Canadians, provide more effective health services and products and strengthen the health care system" [14]. Successful knowledge translation requires turning knowledge into action [13].

A single study is generally not enough evidence for a change in practice, even if it is substantiated by other research results. Clinicians must determine if the results are valid for their own site, since differences can exist between maternity settings. For this reason, we have designed a study protocol for clinicians to use. We are confident that results will be valuable for research-based decision making. In the original study, we showed that if baseline for assessing newborn weight change is changed from birth weight to 24 hours, few newborns lose more than 7% (and only at the 60 and 72 hour measurements) and no newborns lost more than 10% [9]. When the newborn baseline weight is 24 hours, 82% of the newborns regain baseline by Day 7 (n = 91) and 98% return to their baseline weight by Day 12 (n = 88) [9].

## Methods

Prospective data collection, as opposed to a chart audit, permits (or facilitates) more reliable and comprehensive data collection. For some units, the required data are so similar to the hospital records that data can be collected prospectively for all patients who meet the inclusion criteria with little additional paperwork. In our experience, special charting is required. We asked for more frequent newborn weights, output (i.e., diaper) weights which are not routinely measured, and maternal fluid measurements which were outside of what was normally recorded in a patient's chart. For example, with the maternal IV fluids, it was important to stop measuring at birth (i.e., cutting of the cord), but IV totals were usually counted until shift end or when the IV was discontinued. For this protocol, the clinician needs only the amount of fluids given up to the birth, because IV amounts given to women following birth will not affect the neonate.

When designing the study, the first decision is about depth. For some units, undertaking a research study, securing funding, and recruiting participants is a complex and rigorous, but feasible design. For other units, there is a lack of expertise or resources for a full research study, and a simplified monitoring and evaluation program is best.

### Participants

The goal of the protocol is to determine whether prenatal maternal fluids are related to newborn weight loss, and our goal in the original study was to have a representative sample of healthy babies. We suggest inclusion criteria include women expecting a fullterm, single, healthy infant, and planning to breastfeed. Parity is not considered a factor. Birth could be either vaginally or by caesarean section and full term is defined as more than 36 6/7 weeks [15]. In the original study, healthy was defined as able to breastfeed freely and mother and baby being discharged together. For example, women with gestational diabetes or hypertension and infants who were treated for jaundice remained in the study as long as they could breastfeed ad lib and mothers were discharged with their babies. Language requirements must be determined for each site.

Potential participants might be recruited before it has been determined that they meet all of the inclusion criteria. For example, because data must be collected before birth, participants are recruited before the health of the infant has been determined. Additionally, a woman who planned to breastfeed might change her mind following birth. If the baby is not healthy or the mother chooses to not breastfeed, the participant no longer meets inclusion criteria. Supplemented babies remained in the study and were not treated differently from exclusively breastfed babies. Our decision was based on generalizability and evidence that there is little difference in weight loss between exclusively and occasionally supplemented newborns in the first days post partum [9,12]. We recommend running an independent t-test or Mann-Whitney U test to determine whether there is a discernible difference in weight loss between the two groups (exclusively breastfed and supplemented) once data have been gathered.

A variety of recruitment strategies might be used. The key principle is that participants must be identified in time to collect fluid data from admission throughout labour or prior to a caesarean section. The simplest recruitment is during the admission process. An alternate plan is to recruit at prenatal visits to clinicians' offices or prenatal clinics.

Sample size calculations were completed using G*Power [16]. We recommend a sample of 125 mothers which allows for 25-30% attrition. Correlations require a sample of 82 subjects to detect a moderate (.30) correlation at an alpha of 0.05 with a power of 0.80 [16].

### Measurements and data collection

Data collection includes pre and post-birth demographic information, maternal fluids, newborn weights, neonatal output, and infant feeding categories (see Table 1). We recommend data collection at four key points: 1) at registration for the study; 2) from admission to birth; 3) during postpartum period; and 4) at discharge. One must decide if data will be collected by staff or parents. The original study followed participants for 14 days postpartum, and parents took a scale home to continue weight measurements. The researcher followed up with a telephone questionnaire and then picked up the scale from parents.

**Table 1:** Variables

Variable	Collection times	Units	Rationales and notes
Maternal IV fluids	- admission to birth - 6, 8 or 12 hr increments	- millilitres	- millilitres match grams for weight change calculations - match time increments to nursing shifts for reliable data collection - note that admission might be before labour (i.e. starts with induction) - measure to birth only (i.e. when cord is cut), afterwards fluids cannot affect neonate

Maternal IV fluids	- last 2 hours (hrs) before birth	- millilitres	- fluid rebalances over time (fluid in beginning will resettle), so last hours are most significant - final IV fluids positively r/t neonatal output - not certain what timing is most significant, so our decision for two hours is an educated estimate - difficult to guess last hours, but data are important

Maternal oral fluids	- admission to birth - 6, 8 or 12 hr increments	- millilitres	- oral amounts are often considerable (e.g. > 5000 over 12 hours) - significant when combined with IV fluids - oral amounts alone showed no correlation

Newborn weights	- birth then every 12 hrs to 72 hrs or discharge	- weight in grams	- grams match millilitres for weight change calculations - be sure babies are weighed naked - use the same scale - ensure uniformity among scales if more than one used - may need to account for security devices

Neonatal output	- all diapers - 12 hr increments	- weight in grams	- number of diapers is not a reliable measurement - impossible to isolate voids and stools, therefore complete weight of diaper counted as output - weight of the dry diaper must be considered in research design and not added to total weight - 12 hr increments matches the weight measurements and is best for statistical analysis (e.g. to determine if diuresis continues past 24 hrs to 36 hrs - neonates void and stool in utero and at birth before birth weight is measured..We did not try to account for such events - need to account for missed diapers (babies void when diaper is off or stool in the bathtub)

Infant feeding	- birth to 72 hrs or discharge	- yes or no - amount of supplements in mls	- determine if baby is supplemented (yes/no) or if baby is not breastfed - outliers (++ supplements) may need to be removed when cleaning the database - need data on supplementation to determine: 1) if the sample reflects the population and 2) the percentage of weight loss is similar between the supplemented and non-supplemented groups

For planning data collection, make a decision about collecting data after discharge and about who should collect the data. For most sites, restricting data collection up to time of discharge would be most convenient. The post-discharge part of the study was complex and may not be required to answer the key questions about the first two days. Collecting data to discharge (assuming a 48-hour stay) only will allow analysis of the key 24-48 hours in most maternity settings. It will probably not include the nadir of weight loss around 60 hours postpartum.

The registration package should include a patient information sheet (PIS) explaining the study, consent form(s) if they are being used, a contact information sheet, and a prenatal questionnaire (see Table 2). Copies of the PIS, the consent forms, and the instructions for data collection (if parents are collecting data) should be given to the participants to keep. Participants must return signed, dated copies of the consent forms (if they are being used) and the contact information sheet and prenatal questionnaire.

**Table 2:** Forms

Form	Questions	Notes
Prenatal information	1. parity - we counted all children including newborn (i.e. no zeros, although prenatally they are nulliparous) 2. maternal age at birth 3. mom's first language 4. relationship status (committed or not) 5. maternal education 6. family income	- collect data at registration - need description to determine if sample is representative of population

Maternal fluids	- divide into columns for oral and IV fluids - highlight the 2 hr fluids - plan to total amounts before inputting into database	- recommend parents record oral fluids for accuracy - reminder is needed for estimating the last 2 hour-IV amount

Output record	- divide into 12 hour columns with several rows for each diaper weight - note weight of dry diapers - be clear about whether recorded weight includes dry weight of diaper - leave space for missed diapers - plan to add all columns before inputting into database	- use hours (0-12, > 12-24, > 24-36) not day to indicate timing for diapers - participants can confuse first day and second day when the birth time is in between

Weight record	- using 12 hour increments, put one time per line - be clear that weight should be in grams - leave a column for notes - use a column to confirm baby was naked - use a column to indicate any extra weight (e.g. yes/no for security tag) - weight in grams can be entered directly into the database	- timing of weights tend to be simpler than diapers - establish a chart for converting pounds and ounces to grams

Infant feeding	- simple chart to track any supplements - record amount in mls	- prospective records separate from medical records might be more accurate

Postpartum information	1. gestational age 2. infant sex 3. birth type (vaginal or c-section) 4. supplemented (yes/no) 5. amount of supplement	- collect data at discharge when information is known - need description to determine if sample is representative of population - will use supplementation data in analysis

It will be important to determine a procedure to collect data sheets, as data will probably be passed from birth unit to postpartum unit. We recommend colour coding the data collection sheets (e.g. blue paper for fluids, yellow paper for output, green paper for weights). Coloured sheets are a reminder of the study, easily found in the chart, and make it easier when collating and inputting data.

### Data analysis

The amount of maternal fluids, measured in millilitres, is treated as a continuous variable (i.e. zero when the woman did not receive fluids). Baby weights and neonatal output (diaper weights including voids and stools), measured in grams, are also continuous variables, and baby weights could be a negative (i.e., a negative weight loss is actually a weight gain). A conversion chart should be developed in case it is necessary to convert any weights recorded in pounds and ounces.

For ease of use, the protocol has been simplified to 29 key variables, and we have provided an excel file to input data (see Additional File 1). We used SPSS18 and will share a blank SPSS database and the syntax on request. New variables must be created, for example: (a) percentage weight loss from birth; (b) grams of weight loss from birth; (c) percentage weight loss from 24 hours; (d) grams of weight loss from 24 hours; (e) totals of output by hours. For the analysis, one might choose to make weight loss from 36 hours a new variable. We recommend using Shapiro Wilks (a non-parametric one sample test) to determine whether each continuous variable is normally distributed.

Inferential statistics are recommended to test the hypotheses about associations. Pearson's correlation should be used for normally distributed variables. In cases where the variable was not normally distributed, Spearman's rho or Kendall's Tau should be used. Two tailed tests are most commonly used [17]. For convenience, we have provided tables to display results (see Tables 3, 4, 5).

**Table 3:** Newborn weight loss in grams correlated to maternal fluid types (N = ___)

	Type and timing of maternal fluid			
**Timing of** **weight loss**	***IV fluids in last*** ***2 hrs before birth***	***IV fluid*** ***admit to birth***	***Oral fluid*** ***admit to birth***	***All fluids*** ***admit to birth***

Birth to 60 hrs	r = __, p = __ n = __	r = __, p = __ n = __	r = __, p = __ n = __	r = __, p = __ n = __
Birth to 72 hrs	r = __, p = __ n = __	r = __, p = __ n = __	r = __, p = __ n = __	r = __, p = __ n = __

**Table 4:** Maternal fluid amounts correlated to neonatal output (N = ___)

	*Category of maternal fluid *			
**Timing of diaper weight**	***IV fluids in last*** ***2 hrs before birth***	***IV fluids*** ***admit to birth***	***Oral fluids admit to birth***	***All fluids*** ***admit to birth***

0 to 24 hrs	r = __, p = __ n = __	r = __, p = __ n = __	r = __, p = __ n = __	r = __, p = __ n = __
0 to 36 hrs	r = __, p = __ n = __	r = __, p = __ n = __	r = __, p = __ n = __	r = __, p = __ n = __
24 to 48 hrs	r = __, p = __ n = __	r = __, p = __ n = __	r = __, p = __ n = __	r = __, p = __ n = __
48 to 72 hrs	r = __, p = __ n = __	r = __, p = __ n = __	r = __, p = __ n = __	r = __, p = __ n = __

**Table 5:** Percentage of newborn weight loss correlated to neonatal output (N = ___)

	Timing of neonatal output			
**Time of** **weight loss**	***0-24 hours*** ***(n = __ )***	***0-36 hours*** ***(n = __ )***	***24-48 hours*** ***(n = __ )***	***48-72 hours*** ***(n = __ )***

Birth to 24 hours		--	--	--
Birth to 36 hours	--		--	--
24 to 48 hours	--	--		--
48 to 72 hours	--	--	--	

## Discussion

### Ensuring rigour

Controlling for measurement bias is essential, since reliable results require precise measurements. To optimize the gathering of accurate and complete measurement of maternal fluids, infant weight loss, and neonatal output, we recommend that researchers simplify measurement protocols, collect data prospectively when feasible, and ensure equipment is valid and reliable. The same scale should be used for all weights. When more than one scale is used, we recommend checks to ensure that the different scales are in agreement with one another.

When designing the study, we recommend a plan to provide inservices for nurses. Nurses actively participated in our study in both the Birth Units (BU) and the Mother/Baby Units (MBU). Without their participation, data would not have been collected. For quality assurance, it is important that all nurses are well informed. Several strategies were used to inform the nurses. When possible, nurses were included in the initial planning. Their familiarity with routines was an important factor in successful data collection. Initially, nurse managers arranged inservices for the nurses. Posters with a summary of the study were posted and "gentle reminders" were handed out at shift changes.

### Protection of human rights

Ethics approval requirements will vary depending on the type of data collected, the use of the data, and the requirements of each jurisdiction [18]. In some instances, quality assurance appraisals used for assessment or management purposes do not require a research ethics review [18-20]. Decisions about requirements and plans to seek agency and ethics approvals should be made early in the design.

For inquiries requiring research ethics approval, informed consent and protection for privacy and confidentiality are required [18,19]. Consent is needed to collect and use patients' information. In our original study, we had separate consents for the study and for a chart audit. There was controversy about whether one (mother) or both parents should sign the consent for the study [21]. In the end, both parents were asked and space was provided to explain if only the mother signed. The mother signed for the chart audit. We assigned identification numbers and kept the list of identification numbers separated from the participant names in locked cabinets. Only non-identifying information was entered into the database and only aggregated data were reported in publications.

### Other practical issues

We recommend simplifying the protocol. Asking parents to provide the full diaper weight seems simpler than asking them to tare the scale with a diaper on it before weighing the used diaper. The weight of a dry diaper needs to be recorded and then the weight needs to be subtracted from the weight of each used diaper. If more than one type or brand is used, then each dry weight is needed. Either disposable or cloth diapers can be used. Newborns need to be weighed without clothing or diapers. If there is a security tag attached to the newborn, the weight of the tag must be subtracted.

We did not attempt to input missing data regarding weights and fluids, because we could not be certain of the direction (e.g. should weight go up, down, or stay the same?). Babies voided and passed stools when their diapers were off. Parents were asked to document missed output, and we estimated the loss. Parents or staff saved diapers in a plastic bag if they could not be weighed immediately [22].

The study design did not permit precise (i.e. exact millilitre) determination of IV fluids until the cord was cut, nor amounts of insensible loss due to, for example, mucous an infant might have expectorated or fluid exhaled from the lungs. Timing of the first bath or excessive crying might be predictors of weight loss due to additional calories burned, but these data were not collected.

In our original study, we asked for neonatal output (diaper weights) in 24 hour increments. In hindsight, we realized that diuresis might continue for 36 hours, but we did not have the data to test the correlation between the 36 hour weight change and the neonatal output (i.e., impossible to determine amount of output for 36 hours). For this reason, we recommend collecting output in 12 hour increments.

It is important to specify hours for data collection rather than days. For example, it is confusing to say "Day 1" and "Day 2" for a few reasons. Is birth Day 0 or Day 1? When parents whose baby was born on a Tuesday afternoon at 16h00 are asked to collect diapers for Day 1, they may interpret Day 2 to start Wednesday morning rather than on Wednesday after 16 h. Finally, if one assumes birth is Day 1, then one must decide if the Day 1 weight is the birth or 24-hour weight.

For weight, we recommend grams. Grams and millilitres are easily compared. For example 1 litre is considered equal to 1 kilogram; whereas, pounds and ounces do not compare easily with liquid measures.

In the end, the rationale for collecting and analyzing this data is to determine if your clinical setting and birth practices create iatrogenic weight loss that should be considered when assessing breastfed babies. If you, the clinician, find that the neonates experience diuresis in the first 24 (or possibly 36 hours), then you might decide not to use birth weight as a baseline measurement.

## Competing interests

The authors declare that they have no competing interests.

## Authors' contributions

JN-W completed the original study for her doctoral dissertation. AKW supervised the dissertation. JN-W conceived the original study. AKW and DLG contributed to the original observational study design and to interpretation of the results. JN-W wrote the first draft of this manuscript and all authors contributed to the revisions. All authors approved the final manuscript.

## Authors' information

JN-W RN IBCLC PhD is an experienced nurse and lactation consultant who has worked with mothers and their babies in hospital and community settings. JN-W is an assistant professor at the University of Ottawa and the focus of her research program is breastfeeding and human lactation.

AKW RN MSc PhD is dean of the Trent-Fleming School of Nursing. AKW specializes in women's cardiovascular health and quantitative methods.

DLG RN PhD is an epidemiologist in the psychiatry department at Queen's University. DLG specializes in quantitative methods and data analysis.

## Acknowledgements

The authors would like to acknowledge Dr. Wendy Peterson and Dr. William Gibb who contributed to the design and interpretation of the original study. We also acknowledge the colleagues and the parents who participated in the original study.

## Supplementary Material

Additional file 1 Excel file for Data Entry Click here for file
